# Assessing a Myocardial Area at Risk in Non-ST Elevation Acute Myocardial Infarction Without Wall Motion Abnormalities Using Cardiac Magnetic Resonance and Radionuclide Imaging

**DOI:** 10.7759/cureus.55125

**Published:** 2024-02-28

**Authors:** Satoshi Kurisu, Hitoshi Fujiwara

**Affiliations:** 1 Department of Cardiology, Hiroshima-Nishi Medical Center, Otake, JPN

**Keywords:** myocardial metabolism, electrocardiogram, imaging, myocardial injury, myocardial edema

## Abstract

Evaluation of a myocardial area at risk is clinically important because it contributes to clinical decision-making and management of patients with acute myocardial infarction (AMI). Herein, we reported a case of non-ST-elevation AMI (non-STEMI) without wall motion abnormalities on echocardiography, in which the myocardial area at risk was evaluated by two modalities; cardiac magnetic resonance (CMR) and radionuclide imaging. Coronary angiography revealed significant luminal stenosis in the diagonal branch and the obtuse marginal branch. It remained unclear which branch was the culprit. T2-weighted CMR revealed myocardial edema in the left ventricular anterolateral area. Based on the extent of myocardial edema, the patient was diagnosed with non-STEMI in the area corresponding to the diagonal branch. The area exhibiting impaired fatty acid metabolism on iodine-123-beta-methyl-p-iodophenyl penta-decanoic acid (^123^I-BMIPP) imaging matched well with the area showing myocardial edema on T2-weighted CMR. In conclusion, both CMR and BMIPP imaging are powerful tools in identifying a myocardial area at risk even in non-STEMI without wall motion abnormalities. This should contribute to clinical decision-making and management of patients with AMI.

## Introduction

Early reperfusion therapy with percutaneous coronary intervention (PCI), which is the most effective way to limit infarct size, has led to considerable improvement in the survival of patients with acute myocardial infarction (AMI) [1]. However, the final infarct size depends mainly on the extent of the myocardial area related to an occluded coronary artery with a complete absence of blood flow (myocardial area at risk) [2]. Evaluation of a myocardial area at risk is clinically important because it contributes to clinical decision-making and management of patients with AMI.

ST-elevation AMI (STEMI) is a manifestation of complete vessel occlusion due to acute thrombus formation secondary to a ruptured plaque. Characteristically, the ECG (electrocardiogram) shows ST-elevation in the leads corresponding to the occluded coronary artery [3]. Loss of function is one of the characteristics of a myocardial area at risk [4], while the area is usually estimated by transthoracic echocardiography immediately after admission. In contrast, non-STEMI results from incomplete or transient vessel occlusion. The ECG may show ST depression or completely normal [3]. Evaluation of a myocardial area at risk is difficult due to fewer ECG and echocardiographic abnormalities.

Herein, we reported a case of non-STEMI without wall motion abnormalities on echocardiography, in which the myocardial area at risk was evaluated by two modalities: cardiac magnetic resonance (CMR) and radionuclide imaging.

## Case presentation

An 80-year-old woman with hypertension, who had been treated with telmisartan (40 mg) and amlodipine (5 mg) once daily in the morning, presented to the emergency department at night with a three-day history of intermittent chest oppressive sensation. The patient had a history of surgical intervention for right-sided lung cancer three years ago.

On physical examination, her pulse rate was 78 bpm; blood pressure, 142/90 mmHg; body mass index, 21.6 kg/m^2^; oxygen saturation, 98%. There were no audible murmurs. Myocardial enzymes were within normal values, and heart-type fatty acid-binding protein was negative for myocardial injury (Table 1) [5].

**Table 1:** Laboratory data

Variable	Initial presentation	Two days later	Reference range
White blood cell count	8.2 × 10^3^ cells/mm^3^		3.3-8.6 × 10^3^ cells/mm^3^
Red blood cell count	4.11 × 10^6^ cells/mm^3^		3.86-4.92 × 10^6^ cells/mm^3^
Hemoglobin	12.7 g/dL		11.6-14.9 g/dL
Hematocrit	39.7%		35.1-44.4%
Platelet count	215 × 10^3^ cells/mm^3^		158-348 × 10^3^ cells/mm^3^
Total bilirubin	0.52 mg/dL		0.4-1.5 mg/dL
Aspartate aminotransferase	21 U/L	42 U/L	13-30 U/L
Alanine aminotransferase	18 U/L	15 U/L	7-23 U/L
Creatine phosphokinase	155 U/L	239 U/L	41-153 U/L
Creatine phosphokinase-MB	3.9 U/L	12.6 U/L	0-5 U/L
Blood urea nitrogen	19.8 mg/dL	11.6 mg/dL	8-20 mg/dL
Creatinine	0.60 mg/dL	0.59 mg/dL	0.46-0.79 mg/dL
Low-density lipoprotein cholesterol	143 mg/dL		65-163 mg/dL
High-density lipoprotein cholesterol	57.1 mg/dL		48-103 mg/dL
C-reactive protein	0.20 mg/dL		0.00-0.14 mg/dL
Heart-type fatty acid binding protein	Negative		Negative

**Figure 1:**
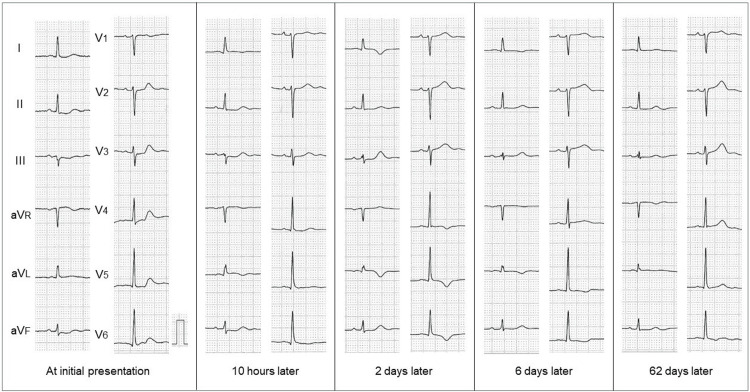
Serial electrocardiograms At the initial presentation, electrocardiography (ECG) revealed no significant ST-elevation in two contiguous leads. However, mild ST depression was seen in leads II, III, aV_^F^_ and V_2-4_. Follow-up ECG showed a newly developed negative T wave in leads I, aV_L_ and V_4-6_.

ECG revealed no significant ST-elevation in two contiguous leads (V_2-3_ leads: >= 1.5 mm; other leads: >= 1 mm). However, mild ST depression was seen in leads II, III, aV_F_, and V_2-4_ (Figure 1). Transthoracic echocardiography showed no left ventricular (LV) wall motion abnormalities with an ejection fraction of 66%. The patient was admitted for further cardiac examinations.

On hospital day 2, although the patient had no recurrent chest symptoms, creatinine kinase (CK) and creatinine kinase-MB (CK-MB) levels were slightly increased to 239 U/L and 12.6 ng/mL, respectively. Follow-up ECG showed newly developed negative T waves in leads I, aV_L_ and V_4-6_ (Figure 1). Follow-up echocardiography failed to detect obvious LV wall motion abnormalities. Given the high possibility of non-STEMI, clopidogrel (75 mg/day) and rosuvastatin (5 mg/day) were initiated.

**Figure 2:**
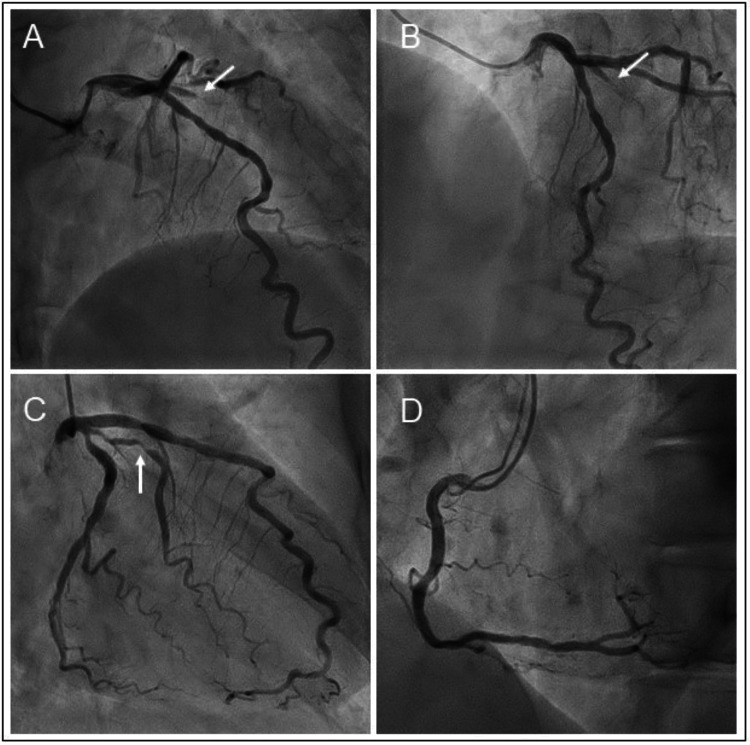
Left and right coronary angiograms Coronary angiography revealed significant luminal stenosis in two separate coronary artery branches; 99% stenosis with thrombolysis in myocardial infarction (TIMI) flow grade 1 in the diagonal branch (A and B, white arrows) and 99% stenosis with TIMI flow grade 2 in the obtuse marginal branch (C, white arrow). No significant stenosis was seen in the major coronary arteries (A to D).

On hospital day 3, coronary angiography was performed, revealing significant luminal stenosis in the following two coronary artery branches: 99% stenosis with thrombolysis in myocardial infarction (TIMI) flow grade 1 in the diagonal branch (Figures 2A-2B, white arrows) and 99% stenosis with TIMI flow grade 2 in the obtuse marginal branch (Figure 2C, white arrow). No significant stenosis was seen in the major coronary arteries (Figures 2A-2D). Because of no obvious wall motion abnormalities on echocardiography, it remained unclear which branch was the culprit.

**Figure 3:**
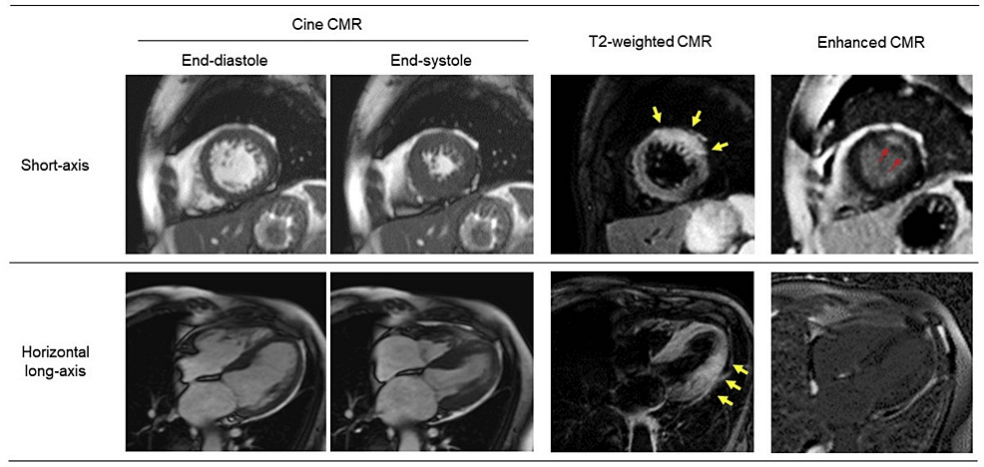
Cardiac magnetic resonance images Cine cardiac magnetic resonance (CMR) revealed no wall motion abnormalities including the area corresponding to the two branches. T2-weighted CMR revealed myocardial edema in the left ventricular anterolateral area (yellow arrows) with subendocardial late gadolinium enhancement (red arrows).

The next day, CMR was performed to identify what myocardial area was at risk (Figure 3). Cine CMR revealed no wall motion abnormalities including the area corresponding to the two branches. These findings were consistent with those on echocardiography. T2-weighted CMR revealed myocardial edema in the LV anterolateral area (Figure 3, yellow arrows) with subendocardial late gadolinium enhancement (Figure 3, red arrows). Based on the extent of myocardial edema visualized by T2-weighted CMR, the patient was diagnosed with non-STEMI in the area corresponding to the diagonal branch. Given the side-branch lesion as well as no symptoms, the coronary intervention was not indicated. Carvedilol (10 mg/day) was additionally initiated.

**Figure 4:**
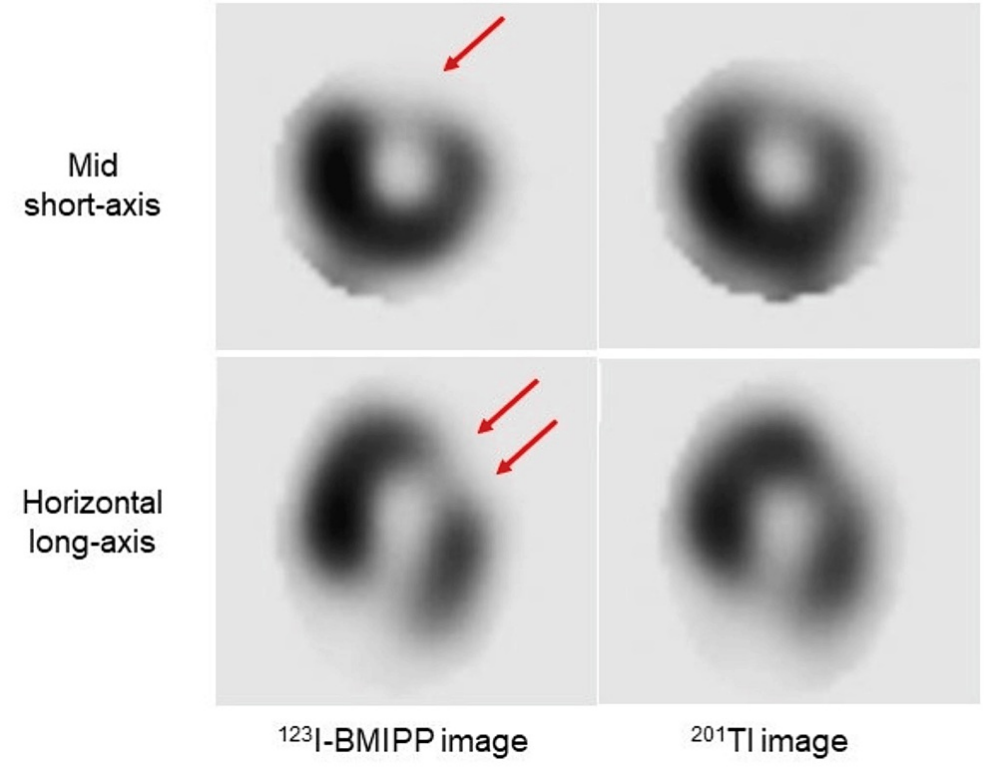
Radionuclide images Single photon emission computed tomography (SPECT) with dual isotopes of thallium-201 (^201^Tl) and iodine-123-beta-methyl-p-iodophenyl penta-decanoic acid (^123^I-BMIPP) was performed. In the left ventricular anterolateral area, fatty acid metabolism was severely reduced on ^123^I-BMIPP imaging (red arrows), whereas the reduction of myocardial perfusion was mild on ^201^Tl imaging. There was an obvious mismatch between ^123^I-BMIPP and ^201^Tl images.

On hospital day 5, single photon emission computed tomography (SPECT) with dual isotopes of iodine-123-beta-methyl-p-iodophenyl penta-decanoic acid (^123^I-BMIPP) and thallium-201 (^201^Tl) was performed (Figure 4). In the LV anterolateral area, fatty acid metabolism was severely reduced on ^123^I-BMIPP imaging (Figure 4, red arrows), whereas the reduction of myocardial perfusion was mild on ^201^Tl imaging. There was an obvious mismatch between ^123^I-BMIPP and ^201^Tl images. The area exhibiting impaired fatty acid metabolism on ^123^I-BMIPP imaging matched well with the area showing myocardial edema on T2-weighted CMR. The patient was treated with conservative management using clopidogrel, rosuvastatin, and carvedilol and was discharged seven days after admission.

Two months later, a follow-up ECG showed the resolution of the negative T waves except for lead V_6_. No abnormal Q waves were present. The patient remained in good condition without recurrent ischemic attacks.

## Discussion

In this report, we presented a case of non-STEMI without wall motion abnormalities, in which both CMR and BMIPP imaging enabled us to identify a myocardial area at risk.

Patients with STEMI typically present with severe ongoing chest pain, thereby they are taken by the ambulance service directly to an emergency center with a review for potential emergency PCI. On the other hand, patients with non-STEMI present more insidiously, and commonly visit a local emergency department [3]. In the present case, the patient was suggestive of non-STEMI after admission based on a combination of clinical history, ECG changes, and cardiac enzymatic release. However, echocardiography revealed no obvious wall motion abnormalities. Coronary angiography showed significant luminal stenosis in two separate coronary artery branches. These modalities failed to identify the extent of the myocardial area at risk and the culprit artery. This was the reason why further cardiac assessment by other imaging modalities was required.

CMR represents a non-invasive imaging modality with increasing applications for the assessment of LV function and perfusion in AMI during a single examination [6,7]. Furthermore, a landmark study by Aletras et al. established the concept that a bright signal on T2-weighted CMR reflects a myocardial area at risk in AMI [8]. In the present case, a unique aspect was the identification of a myocardial area at risk despite the absence of wall motion abnormalities. T2-weighted CMR detected the myocardial area at risk in the anterolateral LV area and allowed us to recognize the diagonal branch was the culprit artery. Arai et al. also recently demonstrated a case of STEMI with triple vessel disease, in which T2-weighted CMR was useful in identifying the culprit artery although no echocardiographic information was provided [9]. CMR contributes to comprehensive cardiac assessment and subsequent management of patients with AMI. Furthermore, recent studies have suggested that the reduction in myocardial edema is a novel target to reduce an irreversible myocardial injury [10]. T2-weighted CMR may serve as a powerful tool to evaluate the effect of anti-myocardial edema strategy.

Another unique aspect was the comparison between CMR and radionuclide imaging [11]. SPECT with ^99m^technesium is an established method for determining a myocardial area at risk. However, this method requires the injection of an isotope before reperfusion with image acquisition within a few hours in an unstable patient situation. This clearly limits the use of SPECT for determining a myocardial area at risk. On the other hand, BMIPP was developed to evaluate regional fatty acid metabolism. Many studies mainly from Japan have reported that BMIPP imaging aids in the determination of a myocardial area at risk even in the subacute phase of AMI [12,13]. BMIPP imaging is an attractive method as an alternative because it can be performed in a stable patient situation. In addition, SPECT with dual isotopes of ^123^I-BMIPP and ^201^Tl aids in the prediction of future cardiac events in patients with AMI, and provides additional predictive value compared with variables obtained by cardiac catheterization alone [14]. This was the reason why SPECT with dual isotopes was further performed after CMR. The mismatch between ^123^I-BMIPP and ^201^Tl images indicates metabolically damaged but viable myocardium, being consistent with subsequent ECG normalization. The abnormal area on ^123^I-BMIPP imaging agreed well with that on T2-weighted CMR. Our findings highlighted that the myocardial area at risk can be visualized as both myocardial edema and impaired fatty acid metabolism after AMI. As for a myocardial injury, CMR was significantly better than ^201^Tl imaging from the viewpoint of the identification of the spatial extent. Further studies are necessary to clarify temporal changes in the extent of myocardial edema and impaired fatty acid metabolism.

## Conclusions

In conclusion, both CMR and BMIPP imaging are powerful tools in identifying a myocardial area at risk even in non-STEMI without wall motion abnormalities. This should contribute to clinical decision-making and management of patients with AMI. In cases of suspected AMI without obvious wall motion abnormalities or adequate echocardiographic image quality, CMR or BMIPP imaging should be considered to identify a myocardial area at risk.
